# Using Videogame Apps to Assess Gains in Adolescents’ Substance Use Knowledge: New Opportunities for Evaluating Intervention Exposure and Content Mastery

**DOI:** 10.2196/jmir.4377

**Published:** 2015-10-28

**Authors:** Erika Montanaro, Lynn E Fiellin, Tamer Fakhouri, Tassos C Kyriakides, Lindsay R Duncan

**Affiliations:** ^1^Yale UniversityNew Haven, CTUnited States; ^2^play2PREVENT Lab at YaleYale School of MedicineNew Haven, CTUnited States; ^3^One Medical GroupSan Francisco, CAUnited States; ^4^Yale School of Public HealthNew Haven, CTUnited States; ^5^play2PREVENT Lab at YaleMcGill UniversityMontreal, QCCanada

**Keywords:** video games, intervention studies, substance use, HIV, evaluation, eHealth

## Abstract

**Background:**

Videogame interventions are becoming increasingly popular as a means to engage people in behavioral interventions; however, strategies for examining data from such interventions have not been developed.

**Objective:**

The objective of this study was to describe how a technology-based intervention can yield meaningful, objective evidence of intervention exposure within a behavioral intervention. This study demonstrates the analysis of automatic log files, created by software from a videogame intervention, that catalog game play and, as proof of concept, the association of these data with changes in substance use knowledge as documented with standardized assessments.

**Methods:**

We analyzed 3- and 6-month follow-up data from 166 participants enrolled in a randomized controlled trial evaluating a videogame intervention, PlayForward: Elm City Stories (PlayForward). PlayForward is a videogame developed as a risk reduction and prevention program targeting HIV risk behaviors (substance use and sex) in young minority adolescents. Log files were analyzed to extract the total amount of time spent playing the videogame intervention and the total number of game levels completed and beaten by each player.

**Results:**

Completing and beating more of the game levels, and not total game play time, was related to higher substance use knowledge scores at the 3- (*P*=.001) and 6-month (*P*=.001) follow-ups.

**Conclusions:**

Our findings highlight the potential contributions a videogame intervention can make to the study of health behavior change. Specifically, the use of objective data collected during game play can address challenges in traditional human-delivered behavioral interventions.

**Trial Registration:**

Clinicaltrials.gov NCT01666496; https://clinicaltrials.gov/ct2/show/NCT01666496 (Archived by WebCite at http://www.webcitation.org/6cV9fxsOg)

## Introduction

In recent decades, eHealth interventions, defined as the use of information technology in the promotion, prevention, treatment, and maintenance of health care [1], have emerged as a new and compelling form of intervention that can reach all ages and ethnic groups [2]. eHealth interventions are a promising form of intervention delivery because they (1) have the potential to reach wider audiences who are not motivated to use in-person approaches, (2) have lower delivery costs than human-delivered interventions, (3) allow for the standardization of delivery content, and (4) offer multiple dissemination channels (eg, smartphones, videogames) [2,3]. Videogames, in particular, are emerging as an effective platform for the delivery of behavioral health interventions [4-6]. For example, Kato et al [7] developed a videogame intervention to address issues related to cancer care and treatment for patients between the ages of 13 and 28 years. After playing either the intervention game or a control game for 1 hour per week for 3 months, patients in the intervention condition had significantly higher self-efficacy and knowledge related to their treatment and management of symptoms as well as greater adherence to medications [7]. This intervention used only videogame play time as an indication of exposure to the intervention. Videogames allow for an interactive experience in a virtual environment, which affords players the opportunity to experience the full spectrum of outcomes related to different choices without actually having to live through the potentially harmful consequences (eg, HIV infection) [8]. Theory-based interventions (ie, those that target and change psychological constructs) are more effective at producing behavior change [9] than non-theory-based interventions and the advancement of videogame interventions and technology in general allows researchers to better understand the entire process.

### Active Ingredients of Change

Interventions designed from health behavior theory are more successful than those that are not theory-based [9,10]. Behavior change interventions are often complex, targeting multiple theoretical constructs in hopes of changing behavior [11]. The complex framework in which many interventions are built means there are a variety of factors being intervened on at once; thus, a limitation of conventional behavior change interventions is the ability to accurately catalog exposure to a given intervention component. Because behavioral interventions are now delivered via emerging technologies, opportunities to improve on traditional assessments of intervention implementation are being established [12]. Indeed, for more than a decade, researchers have been encouraged to think creatively to establish systems for gathering data through these eHealth interventions to explore the active ingredients of change [13,14]. Videogame-based interventions provide a concrete example of how innovative techniques to measure exposure can be implemented through eHealth interventions.

### Importance of Implementation Guides

Videogames have the advantage of providing highly accurate and granular exposure to information through the analysis of each player’s unique game play and decisions. Videogame software can create automatic logs with timestamps of all aspects of a player’s experience (eg, button presses, game levels entered and exited, actions taken) to a level of detail allowing for the recreation of the entire game play session. New avenues for data collection using videogames, and broadly all Internet-delivered interventions, are emerging [11]. Implementation guides for these new assessment methods, such as using event log files, are needed to catalog these techniques in a purposeful, coherent, and understandable way to advance the field [15]. These guides will allow for the level of detail needed to create new research questions regarding game play interactions, participant experience, and process evaluation.

### Substance Use

Adolescents use substances such as alcohol and marijuana. In 2011, 70% of students in grade 12 reported having tried alcohol and 50% reported being drunk at least once [16]. Marijuana is another substance used frequently in adolescence with 46% of those in grade 12 reporting using marijuana at least once in their lifetime [16]. Substance use is often associated with unprotected sexual activities [17,18] and an increased number of accidental deaths among adolescents [19]. However, it is unclear if adolescents are knowledgeable about the negative consequences related to substance use. It is critically important to examine what adolescents know about substance use to create effective risk reduction interventions.

### This Study

This study acts as a guide, demonstrating (1) the analysis of automatic log files created by videogame software that catalog game play and (2) the association of these data with changes in substance use knowledge documented by assessments. This videogame-based intervention was grounded in social cognitive theory [20] and message framing [21]—derived from prospect theory [22]—and was developed to reduce HIV risk behaviors among young adolescents. The purpose of this paper is to describe how a technology-based intervention can yield meaningful, objective evidence of intervention exposure and participant experience within a behavioral intervention. As proof of concept, we will then demonstrate how these data relate to an important psychological construct related to behavioral outcomes: knowledge [23]. We sought to determine how performance in the game (eg, by points earned) relates to knowledge as measured using a standardized validated instrument. The implications of this strategy for extracting data from the game software and evaluating participants’ game processes within a behavioral intervention can be extrapolated to interventions employing a wide variety of other technologies. Utilizing log files to capture a player’s unique game play decisions and process will ultimately facilitate a broader understanding of behavior change and potentially support the use of videogames not only as effective interventions, but also as valid assessment tools.

## Methods

### Participants

A total of 333 participants, aged 11 to 14 years, were enrolled in a randomized controlled trial in which they played either an intervention game, *PlayForward: Elm City Stories*, or a set of attention- and time-control games for a maximum of 16 hours over 6 weeks. Participants were recruited from community afterschool, school, and summer programs in New Haven, CT. All procedures were approved by the Yale University Human Investigation Committee.

### The Videogame Intervention

*PlayForward: Elm City Stories* is a videogame developed as a risk reduction and prevention program targeting HIV risk behaviors, primarily sex- and substance use–related, in young minority adolescents. *PlayForward* was developed through extensive formative work with the target audience [5,8,24,25]. The main storyline of the game is comprised of “challenge stack” levels in which players travel through a virtual life from grade 7 to 12 and engage in role-playing scenarios where they must make decisions around risky behaviors (eg, unprotected sex, alcohol use) and experience the positive and negative consequences of those behaviors. The participants acquire risk-related knowledge, navigate peer relationships, and negotiate against peer pressure. Players encounter realistic stories experienced by middle school and high school students, such as sneaking into a significant other’s house, unplanned pregnancy, vandalism, and drunk driving. Players must also earn points in mini-games designed to build knowledge or behavioral skills needed to avoid risk, such as refusal, negotiation, or peer-assessment skills. Through these mini-games, players acquire the “senses” and “powers” needed to resolve the stories (for a complete list of the mini-games see [24]). To demonstrate our analysis strategy, we will focus on *Know Power*.

*Know Power* provides the player with accurate and relevant information about the consequences of engaging in risky behaviors. This mini-game, comprised of 10 levels, emphasizes the development and clarification of adolescents’ values and evaluations of risk behavior and provides them with information about the consequences of and alternatives to engaging in these behaviors. Positive attitudes toward avoiding substance use, delaying sexual initiation, and general risk reduction are cultivated in this component of the game. *Know Power* specifically focuses on increasing knowledge and creating positive attitudes about HIV risk reduction behaviors.

### Measures

#### Knowledge

Participants were asked to complete a 22-item multiple-choice assessment of substance use and sexual health knowledge at each time point. The 22 items related to knowledge were adapted from several adolescent knowledge content sources, including an evidence-based curriculum that has been proven effective in reducing the risk of HIV, sexually transmitted infections, and teen pregnancy in young minority adolescents [26-28]. For this study, the subset of 8 questions addressing substance use knowledge was used. Participant response options were true, false, and not sure. These items included:

1. Drug users who use needles to inject drugs into their bodies have a greater chance of getting HIV if they share needles with other people.
2. People who use drugs occasionally can’t become addicted to them.
3. Taking someone else’s prescription drugs is safe as long as the person giving them to you says it is okay.
4. Using any drug can be more dangerous when taken with alcohol or other drugs.
5. People are more likely to make unsafe decisions about sex (eg, not wearing a condom) if they have been using drugs or drinking alcohol.
6. Drinking coffee or taking a cold shower can help sober someone up who is drunk.
7. If a person drinks too much alcohol, they might get sick from alcohol poisoning.
8. Alcohol poisoning can cause a person to stop breathing or choke to death on their own vomit.

#### Game Play Log Files

*PlayForward* was designed to capture granular information about each player’s gameplay experience, actions, and behavior in event logs. There were 2 kinds of data collected: (1) player game state data, the traditional save/load data required so that a player can save their progress and then pick up where they left off later and (2) activity logging data that captures relevant actions players took during game play. Activity logging data included which game content players were exposed to, how long it took them to solve particular game levels, how much time was spent playing various portions of the game, when players beat particular levels of the game, and how many levels were beaten. Every action that the player performed in the game-selecting options, entering/exiting a game area, or making a choice was recorded with a timestamp. Timestamped data were stored in comma-delimited log files, which were then imported into a database for data analysis. Two game play variables from the activity logging data were extracted: the total amount of time spent playing *PlayForward* and the total number of game levels (challenge stacks) beaten. Total game play time was calculated by identifying timestamped game start and end events, calculating a time interval for each game session, and then summing the time across all play sessions during the trial. Total number of game levels beaten was calculated by adding up the number of game level completion events for each player. We did not focus on which game content players were exposed to because *PlayForward* is sequenced in a linear fashion, such that players are required to beat particular mini-game levels in a specific sequence to progress through challenge stacks. Therefore, players with the same number of total levels beaten were exposed to the same content.

Players in the *PlayForward* cohort were divided into 2 subsets (high scoring and low scoring) based on whether they were above (high scoring) or below (low scoring) the median number (median=8) of total game levels beaten. When participants were instructed to restart the game, game play time from their second attempt was factored into the total number of hours played, but completion of game levels a second time did not count toward a higher score (scores were capped at 12 because there are 12 levels in the game).

### Data Analysis

Substance use knowledge was examined for participants in the *PlayForward* condition at baseline, 3 months, and 6 months postintervention. First, a repeated measures ANOVA was used to examine mastery effects on substance use knowledge over time. Bivariate relationships were also examined among log file variables and substance use knowledge. A 2-sample *t* test was used to compare substance use knowledge scores between the high- and low-scoring groups at each time point. Multivariate regression methods were used to assess the impact of baseline knowledge score, total amount of time spent playing *PlayForward*, and total number of game levels (challenge stacks) beaten by each player on the gain in substance use knowledge at 3- and 6-months postintervention.

## Results

For the current study, only participants in the experimental condition (ie, *PlayForward*) were examined because these were the participants with game play data available (N=166; mean age 12.95, SD 1.03 years; mean game play time 7.27, SD 3.55 hours; median 8.24, IQR 3.70-10.14 hours; see Table 1 for demographics). Of the participants in the *PlayForward* group, 72 were in the high-scoring group, 72 were in the low-scoring group, and 22 players either had missing or corrupted log file data due to game software or transcription errors. Those 22 players with missing or corrupted log files were not included in the analyses. There was a significant difference between the age groups such that older participants were also more likely to be high-scoring players. A total of 37 players beat the *PlayForward* videogame and were instructed to restart the game and play through a second time.

**Table 1:** Baseline demographic characteristics of participants (N=144).

Characteristic		Group by game play score		Test statistic		*P*
		High score n=72	Low score n=72	χ^2^ (*df*)	*t*_142_	
Gender (female), n (%)		36 (50)	31 (43)	0.7 (1)		.40
Age (years), mean (SD)		13.18 (1.03)	12.72 (1.03)		2.68	.01
**Race/Ethnicity, n (%)**				0.6 (4)		.96
	Caucasian	3 (4)	3 (4)			
	Black	27 (38)	23 (32)			
	Hispanic	25 (35)	29 (40)			
	Biracial	8 (11)	8 (11)			
	Other	9 (13)	9 (13)			

### Group Differences in Substance Use Knowledge

Knowledge scores were assigned based on the total number of correct answers on the test (maximum score=8; mean 4.52, SD 1.81). There were no statistically significant differences between the groups with respect to baseline substance use knowledge score (high scoring: mean 4.65, SD 1.80; low scoring: mean 4.42, SD 1.82; *t*_102_=–0.64, *P*=.52). Players with a high score in the game had significantly higher scores on the assessment of substance use knowledge at both 3-month (high scoring: mean 6.72, SD 1.45; low scoring: mean 4.74, SD 2.42; *t*_102_=–5.11, *P*=.001) and 6-month (high scoring: mean 6.56, SD 1.53; low scoring: mean 4.78, SD 2.38; *t*_102_=–4.57, *P*=.001) follow-ups. Specifically, players who mastered the intervention material (ie, high-scoring players) were also more knowledgeable of substance use facts (eg, “If a person drinks too much alcohol they might get sick from alcohol poisoning”). A significant mastery by time interaction existed for substance use knowledge (*F*_2,101_=9.41, *P*=.001). High-scoring players had greater substance use knowledge at the 3- and 6-month follow-up periods.

### Factors Associated with Substance Use Knowledge

We examined the bivariate relationships among the log file data (number of levels beaten: mean 7.64, SD 3.61; number of hours playing game: mean 7.27, SD 3.55) and substance use knowledge at baseline (mean 4.52, SD 1.81) and at the 3-month (mean 5.68, SD 2.24) and 6-month (mean 5.65, SD 2.18) follow-ups (see Table 2). Baseline substance use knowledge was not associated with either number of levels beaten or total time spent playing the game. Both 3- and 6-month substance use knowledge were positively associated with number of levels beaten or total time spent playing the game. Number of levels beaten was more strongly related to 3- and 6-month substance use knowledge than total time spent playing the game.

**Table 2:** Relationship between knowledge and game play variables.

Knowledge and game play	1		2		3		4		5	
	*R* ^2^	*P*	*R* ^2^	*P*	*R* ^2^	*P*	*R* ^2^	*P*	*R* ^2^	*P*
1. Baseline knowledge^a^	__									
2. 3-Month knowledge^a^	.411	.001	—							
3. 6-Month knowledge^a^	.331	.001	.809	.001	—					
4. Number of levels beaten^b^	.113		.528	.001	.531	.001	—			
5. Number of hours playing game	–.033		.205	.03	.219	.02	.584	.001	—	

^a^ Knowledge scores range from 0 to 8.

^b^ Number of levels beaten maximum=12.

### Predictors of Substance Use Knowledge at Follow-Up

#### Three-Month Follow-Up

Age differences were found at baseline (Table 1) such that older participants were more likely to also be high-scoring players; therefore, age was included in the model to determine if age differences were responsible for mastery of game material. Collapsing across groups (ie, high scoring vs low scoring), results of the multivariate regression analyses examining game play variables as predictors of substance use knowledge were significantly related to substance use knowledge at the 3-month follow-up (Table 3) (*R* ^2^=.396, *F*_4,102_=18.36, *P*=.001). Only 2 predictors were significantly uniquely associated with substance use knowledge at 3 months; baseline knowledge (*B=*.281, *t*_106_=3.47, *P*=.001) and game levels beaten (*B=*.489, *t*_106_=5.12, *P=*.001) were positively related to substance use knowledge at 3 months. Higher scores on these constructs were associated with higher substance use knowledge at 3 months. Age and total time playing *PlayForward*, on the other hand, were not statistically significantly related to substance use knowledge at 3 months.

**Table 3:** Association of game play with gains in substance use knowledge.

Substance use knowledge		*B*	SE	β	*t*_102_	*P*
**3 Month**						
	Number of levels beaten	.31	0.06	.49	5.12	.001
	Number of hours playing game	–.07	0.07	–.09	–1.00	.32
	Baseline knowledge	.35	0.10	.28	3.47	.001
	Age	.38	0.18	.16	1.85	.07
**6 Month**						
	Number of levels beaten	.11	0.05	.18	2.19	.03
	Number of hours playing game	–.02	0.05	–.03	–0.35	.71
	3-month knowledge	.72	0.07	.74	10.23	.001
	Age	–.13	0.13	–.06	–0.98	.33

#### Six-Month Follow-Up

Similar patterns appeared for substance use knowledge at the 6-month follow-up (Table 3) (*R* ^2^=.644, *F*_4,102_=48.97, *P*=.001). Only 2 predictors were significantly uniquely associated with substance use knowledge at 6 months; 3 month knowledge (*B=*.735, *t*_106_=10.23, *P=*.001) and game levels beaten (*B=*.179, *t*_106_=2.19, *P=*.03) were positively related to substance use knowledge at 6 months. Higher scores on these constructs were associated with higher substance use knowledge at 6 months. Age and total time playing *PlayForward*, on the other hand, were not statistically significantly related to substance use knowledge at 6 months. Overall, it was number of levels beaten (ie, mastery of intervention content) that was associated with a gain in substance use knowledge at 3 months and 6 months.

## Discussion

This study describes how a videogame intervention can produce objective data demonstrating exposure to the intervention and yield meaningful evidence of the association between a participant’s success within the videogame and changes in an important psychological construct—knowledge. We examined how 2 separate measures of game play—total amount of time spent playing the *PlayForward* videogame and the total number of game levels (challenge stacks) beaten by each player—might be related to substance use knowledge at 3- and 6-month follow-ups. These 2 measures of game play broaden the scope of traditional face-to-face intervention assessment by accurately tracking the participant’s success within the game. For example, it appears that mastery of the intervention material (eg, game levels beaten), and not time spent playing each level, influences acquisition of substance use knowledge.

High-scoring game players gained and retained significantly more knowledge about substance use at the follow-up time points than their low-scoring counterparts did. Interestingly, completing and beating more of the game levels, and not total game play time, resulted in greater changes in knowledge over time. Mastery of the material increases substance use knowledge for up to 6 months after game play has been completed.

### Implications for eHealth Interventions

There is a growing body of literature demonstrating eHealth interventions as efficacious tools for creating behavior change [12,29]. However, it is still largely unknown *how* this change occurs. The current study examined one behavioral antecedent of behavior change—knowledge—to illustrate how objective data collected throughout game play can contribute to our understanding of how change happens. An implicit assumption in recommendations for behavior change interventions is that more time spent in interventions will produce the greatest amount of behavior change [30]. For example, an intervention examining the effects of a single-session versus a multiple-session smoking cessation intervention suggests multiple sessions (ie, more time spent in the intervention) are related to significantly higher abstinence rates than a single session [31]. Our data, on the other hand, suggest that it is not time but mastery of the material that is related to changes of important psychological mediators (ie, knowledge) of behavior change. This is a valuable methodological point. Many eHealth interventions merely present information to participants [13], much like a brochure, but do not include content that must be mastered. Our data suggest that simple exposure to intervention content is not enough to create change; instead, what is needed is the opportunity to process intervention content more deeply. It is not enough to give people information, we must give them a means by which to master intervention content to change behavior.

Traditional human-delivered interventions rely heavily on self-report data or a measure of overall intervention time as a proxy measure to examine an individual participant’s experience throughout an intervention. eHealth interventions, such as videogames, allow researchers to objectively track a participant’s intervention experience through the game software by easily collecting data on time spent in individual segments of the intervention, overall time spent completing the intervention, and mastery of intervention material. This information can then be used to learn which material is most important to the behavior change process. For example, the data collection features of eHealth interventions could allow us to determine if one portion of an intervention dedicated to knowledge acquisition mastery is enough to produce knowledge gains at follow-up interval periods or if more sessions are necessary. This is just one of the questions that can be answered by using a technology-based intervention as a data collection tool. Before eHealth interventions, these questions were burdensome and resource-intensive to investigate [32]. These are empirical questions that can—and should—be answered to facilitate optimal intervention development and to inform the progress of theoretical innovations in the behavior change domain.

Behavior change interventions target multiple constructs at once to create behavior change [11]. Thus, conventional behavior change interventions are unable to determine which intervention components are contributing to behavior change. A dismantling study design could help determine if a focused manipulation of one construct can have diffuse effects on other constructs [33] and/or if such a focused manipulation of one construct is as good as or better than manipulation of multiple constructs simultaneously. Given the lower delivery cost, standardization of delivery content, multiple dissemination channels, and objective data collection of participant experience [2,3], eHealth interventions are in a unique and prime position to answer these questions.

### Limitations

With this study, as a demonstration of the novel methods of evaluating gameplay data, we chose to examine the behavioral antecedent of knowledge, not behavioral outcomes, which will be included in future analyses. Another limitation of this study was that knowledge relied on a self-report measure; this limitation is shared with the majority of intervention research. However, log file data offer unique assessment techniques to examine intervention exposure and outcomes whereby adding another valid source of data and potentially reducing the need to rely solely on self-report data. Additionally, only a small number of items (ie, 8) were used to assess substance use knowledge.

### Conclusions

Increasingly, eHealth interventions, particularly videogames, are becoming a standard and effective form of intervention delivery [2-5]. Interventions delivered via emerging technologies have the potential to enhance traditional forms of assessment with real-time objective data collection techniques. Importantly, videogames can and do serve a dual purpose of delivering health interventions and providing a unique assessment tool with minimal additional effort required from participants. Participant log files are just one potential technique. Our findings highlight the valuable contributions eHealth interventions make to the study of health behavior change by using objective data collection techniques that are difficult to replicate in traditional human-delivered behavioral interventions. This research illustrates the need for the rigorous exploration of new data collection opportunities provided by eHealth interventions. These innovative methods hold the potential to document specific engagement, interactions, and exposure that may provide key information regarding which intervention components are most effective in influencing participant outcomes.

## References

[1] Eysenbach G (2001). What is e-health?. J Med Internet Res.

[2] Lehto T, Oinas-Kukkonen H (2011). Persuasive features in web-based alcohol and smoking interventions: A systematic review of the literature. J Med Internet Res.

[3] Noar SM, Black HG, Pierce LB (2009). Efficacy of computer technology-based HIV prevention interventions: A meta-analysis. AIDS.

[4] Hieftje K, Edelman EJ, Camenga DR, Fiellin LE (2013). Electronic media-based health interventions promoting behavior change in youth: A systematic review. JAMA Pediatr.

[5] Fiellin LE, Hieftje KD, Duncan LR (2014). Videogames, here for good. Pediatrics.

[6] Ruggiero L, Moadsiri A, Quinn LT, Riley BB, Danielson KK, Monahan C, Bangs VA, Gerber BS (2014). Diabetes island: Preliminary impact of a virtual world self-care educational intervention for African Americans with type 2 diabetes. JMIR Serious Games.

[7] Kato PM, Cole SW, Bradlyn AS, Pollock BH (2008). A video game improves behavioral outcomes in adolescents and young adults with cancer: A randomized trial. Pediatrics.

[8] Hieftje K, Duncan LR, Fiellin LE (2014). Novel methods to collect meaningful data from adolescents for the development of health interventions. Health Promot Pract.

[9] Glanz K, Bishop DB (2010). The role of behavioral science theory in development and implementation of public health interventions. Annu Rev Public Health.

[10] Noar SM (2008). Behavioral interventions to reduce HIV-related sexual risk behavior: Review and synthesis of meta-analytic evidence. AIDS Behav.

[11] Norman GJ, Zabinski MF, Adams MA, Rosenberg DE, Yaroch AL, Atienza AA (2007). A review of eHealth interventions for physical activity and dietary behavior change. Am J Prev Med.

[12] Riley WT, Rivera DE, Atienza AA, Nilsen W, Allison SM, Mermelstein R (2011). Health behavior models in the age of mobile interventions: Are our theories up to the task?. Transl Behav Med.

[13] Strecher V (2007). Internet methods for delivering behavioral and health-related interventions (eHealth). Annu Rev Clin Psychol.

[14] Neuhauser L, Kreps GL (2003). Rethinking communication in the e-health era. J Health Psychol.

[15] Barak A, Klein B, Proudfoot JG (2009). Defining internet-supported therapeutic interventions. Ann Behav Med.

[16] Johnston L, O’Malley P, Bachman J, Schulenberg J (2012). Monitoring the Future National Survey Results on Drug Use, 1975–2011. Volume I: Secondary School Students.

[17] Weinhardt LS, Carey MP (2000). Does alcohol lead to sexual risk behavior? Findings from event-level research. Annu Rev Sex Res.

[18] Anderson BJ, Stein MD (2011). A behavioral decision model testing the association of marijuana use and sexual risk in young adult women. AIDS Behav.

[19] Eaton D, Kann L, Kinchen S, Shanklin S, Flint KH, Hawkins J, Centers for Disease Control and Prevention (2012). Youth risk behavior surveillance—United States, 2011. MMWR Morb Mortal Wkly Rep.

[20] Bandura A (1998). Health promotion from the perspective of social cognitive theory. Psychol Health.

[21] Rothman AJ, Salovey P (1997). Shaping perceptions to motivate healthy behavior: The role of message framing. Psychol Bull.

[22] Kahneman D, Tversky A (1979). Prospect theory: An analysis of decision under risk. Econometrica.

[23] Fisher JD, Fisher WA, Misovich SJ, Kimble DL, Malloy TE (1996). Changing AIDS risk behavior: Effects of an intervention emphasizing AIDS risk reduction information, motivation, and behavioral skills in a college student population. Health Psychol.

[24] Duncan LR, Hieftje KD, Culyba S, Fiellin LE (2014). Game playbooks: Tools to guide multidisciplinary teams in developing videogame-based behavior change interventions. Transl Behav Med.

[25] Hieftje K, Rosenthal MS, Camenga DR, Edelman EJ, Fiellin LE (2012). A qualitative study to inform the development of a video game for adolescent HIV prevention. Games Health J.

[26] Jemmott JB, Jemmott LS, Fong GT (2010). Efficacy of a theory-based abstinence-only intervention over 24 months: A randomized controlled trial with young adolescents. Arch Pediatr Adolesc Med.

[27] Kelly JA, St Lawrence JS, Hood HV, Brasfield TL (1989). An objective test of AIDS risk behavior knowledge: scale development, validation, and norms. J Behav Ther Exp Psychiatry.

[28] Jemmott L, Jemmott J, McCaffree K (2002). Making a Difference!: An Abstinence Approach to HIV/STD and Teen Pregnancy Prevention.

[29] Webb TL, Joseph J, Yardley L, Michie S (2010). Using the internet to promote health behavior change: A systematic review and meta-analysis of the impact of theoretical basis, use of behavior change techniques, and mode of delivery on efficacy. J Med Internet Res.

[30] Peters E, Klein W, Kaufman A, Meilleur L, Dixon A (2013). More is not always better: intuitions about effective public policy can lead to unintended consequences. Soc Issues Policy Rev.

[31] Zhu SH, Stretch V, Balabanis M, Rosbrook B, Sadler G, Pierce JP (1996). Telephone counseling for smoking cessation: Effects of single-session and multiple-session interventions. J Consult Clin Psychol.

[32] Jacobson NS, Dobson KS, Truax PA, Addis ME, Koerner K, Gollan JK, Gortner E, Prince SE (1996). A component analysis of cognitive-behavioral treatment for depression. J Consult Clin Psychol.

[33] Borkovec TD, Newman MG, Pincus AL, Lytle R (2002). A component analysis of cognitive-behavioral therapy for generalized anxiety disorder and the role of interpersonal problems. J Consult Clin Psychol.

